# Perspectives on Episodic-Like and Episodic Memory

**DOI:** 10.3389/fnbeh.2013.00033

**Published:** 2013-04-18

**Authors:** Bettina M. Pause, Armin Zlomuzica, Kiyoka Kinugawa, Jean Mariani, Reinhard Pietrowsky, Ekrem Dere

**Affiliations:** ^1^Institute of Experimental Psychology, University of DüsseldorfDüsseldorf, Germany; ^2^Center for the Study and Treatment of Mental Health, Ruhr-Universität BochumBochum, Germany; ^3^Neurobiologie des Processus Adaptatifs-UMR 7102, Université Pierre et Marie CurieParis, France; ^4^UMR 7102, CNRSParis, France; ^5^Institut de la longévité, AP-HP Hôpital Charles FoixIvry-sur-Seine, France

**Keywords:** Alzheimer disease, dissociative disorders, emotional memory, episodic memory, mild cognitive impairment, spatial memory, temporal order memory, test development

## Abstract

Episodic memory refers to the conscious recollection of a personal experience that contains information on what has happened and also where and when it happened. Recollection from episodic memory also implies a kind of first-person subjectivity that has been termed autonoetic consciousness. Episodic memory is extremely sensitive to cerebral aging and neurodegenerative diseases. In Alzheimer’s disease deficits in episodic memory function are among the first cognitive symptoms observed. Furthermore, impaired episodic memory function is also observed in a variety of other neuropsychiatric diseases including dissociative disorders, schizophrenia, and Parkinson disease. Unfortunately, it is quite difficult to induce and measure episodic memories in the laboratory and it is even more difficult to measure it in clinical populations. Presently, the tests used to assess episodic memory function do not comply with even down-sized definitions of episodic-like memory as a memory for what happened, where, and when. They also require sophisticated verbal competences and are difficult to apply to patient populations. In this review, we will summarize the progress made in defining behavioral criteria of episodic-like memory in animals (and humans) as well as the perspectives in developing novel tests of human episodic memory which can also account for phenomenological aspects of episodic memory such as autonoetic awareness. We will also define basic behavioral, procedural, and phenomenological criteria which might be helpful for the development of a valid and reliable clinical test of human episodic memory.

## Introduction

Learning and memory are indispensable capacities for humans and animals, since they permit adaptive behavior and promote the survival of the individual and the species. For example, they allow animals to revisit places where food or mating resources can be found and to avoid places where odor trails of predators were present. In general, they allow flexible and adaptive behavior in response to slow or sudden changes in the environment. The importance of learning and memory for the everyday life in humans becomes evident when one considers the decomposed personality structure in people who have lost access to information about emotionally relevant life events, such as in the case of demented patients.

Clinical studies with brain-injured patients and lesion studies in animals have revealed multiple memory systems in the brain with distinct neuroanatomical substrates and which are specialized for the learning of specific material such as how to play piano or the contents of a textbook (Squire, 2004). Accordingly, long-term memories can be divided into declarative and non-declarative memories. Declarative or explicit memories are conscious, can be voluntarily accessed and can be verbalized. In contrast non-declarative memories are not conscious and the contents of these memories cannot be verbalized. Declarative memories can be further subdivided into semantic and episodic memories. Semantic memories refer to facts and rules and basic knowledge about the world (Squire, 2004). In contrast, episodic memories refer to single events or personal experiences that also contain information about the spatial and temporal context of these events. Episodic memories also contain a blueprint of the internal state of the individual during encoding, e.g., its emotions, perceptions, and thoughts (Dere et al., 2008, 2010).

Due to its complexity of being a multi-dimensional memory trace that is distributed across the central nervous system and since it is established on a single occasion, episodic memory is highly vulnerable to disease conditions and easily disturbed (Aggleton and Brown, 1999; Aggleton and Pearce, 2001). Impairments in episodic memory function are observed in individuals with Mild Cognitive Impairment (MCI), in neurodegenerative diseases such as Alzheimer’s Disease (AD), Huntington’s Disease (HD), and Parkinson’s Disease (PD) and also in a number of psychiatric diseases including Schizophrenia, Major Depression (MD), and dissociative disorders.

In this review we will describe the concept of episodic memory, and present human disease conditions that are associated with episodic memory impairment. In the main part of this review, we will describe currently used tests of episodic memory function and discuss their validity. Hereby, we will discuss the implications of animal research on episodic-like memory for the theory and measurement of episodic memory. We will also describe a new concept of episodic memory that addresses the important questions of what is actually triggering episodic memory formation and its retrieval, and why some events are stored only transiently and others permanently. Finally, we will define basic criteria for the development of valid tests of episodic-like memory.

## Endel Tulving’s Concept of Episodic Memory

The concept of episodic memory was developed by Endel Tulving in the early 70s (Tulving, 1972, 1983). At this time Tulving defined episodic memory rather technically as a memory system specialized to store specific idiosyncratic experiences in terms of what happened and where and when it happened. This initial definition is amenable to operationalization using a pure behaviorist approach to measure learning and memory performance (Dere et al., 2008, 2010) and has stimulated the development of what, where, and when paradigms for testing whether animals have the capacity to form episodic memories (Clayton and Dickinson, 1998; Dere et al., 2005a; Roberts et al., 2008).

In later work, Tulving widened the concept of episodic memory to include prerequisites of a fully developed episodic memory system (Tulving, 2001, 2002). Additionally, he described phenomenological processes that are specifically associated with the retrieval of episodic but not semantic memories. According to Tulving, episodic memory depends on a *self* (the awareness of the own existence) that goes along with *autonoetic awareness* (the awareness that remembered personal experiences have happened to oneself, are not happening now, and are part of one’s personal history). Furthermore, Tulving proposed that humans have a *sense of subjective time* which enables them to distinguish between mental representations of the *self* in the past, present, and future (Tulving, 2001, 2002). Recently, the definition of episodic memory has been expanded by Klein (2013; this issue) by postulating that the core features of episodic memory in terms of a memory for what, happened, where and when are also shared by semantic memory and that episodic recollection requires the coordinated function of a number of distinct, but interacting, “enabling” systems. As enabling systems Klein postulates the para-mnestic constructs “ownership,” “self,” “subjective temporality,” and “agency” that are necessary for episodic or autonoetic recollection in the sense of Tulving (2001, 2002).

It is further assumed that the type of subjective awareness provided by episodic or autonoetic recollection is relational rather than intrinsic in nature. Klein also assumes that the latter would imply that in some patient populations autonoetic recollection can be disturbed while core elements of episodic-like memory remain intact and are indistinguishable from the content of semantic long-term memory (Klein, 2013).

Nevertheless, it is obvious that the assessment of these phenomenological aspects during the retrieval of episodic memories is difficult to obtain by objective methods (Pennartz, 2009; Tagini and Raffone, 2010; Aleksander and Gamez, 2011). The possibilities to develop tests of autonoetic awareness and episodic recollection will be discussed later in this article. In the second part of this review we will outline the advantages of a behavioral definition of episodic-like memory for the development of a test that can be used for diagnostic purposes in healthy and patient populations and that allows to measure whether at least episodic-like memory is intact. We will also discuss the possibilities to combine a test of episodic-like memory with a second test that measures phenomenological aspects of episodic memory including autonoetic awareness to capture all elements of human episodic memory.

## Impairments of Human Episodic Memory

Episodic memory deficits are observed after medial temporal lobe injury (Nyberg et al., 1996) which includes important memory structures such as the hippocampus (Burgess et al., 2002) and amygdala (Markowitsch and Staniloiu, 2011), but also after lesions to the frontal cortex (Kirchhoff et al., 2000) and diencephalic structures, such as the mediodorsal thalamus and the mammillary bodies (Tsivilis et al., 2008; Wolff et al., 2008).

Episodic memory impairments have also been demonstrated in the course of healthy aging (Tulving, 1983; Shing et al., 2010), the acute phase following mild traumatic brain injury (Dickerson and Eichenbaum, 2010; Tsirka et al., 2010), and in a variety of neuropsychiatric diseases (Butters et al., 1987; Dere et al., 2010). Furthermore, it seems that episodic memory deficits usually precede more global cognitive impairments associated with neurodegenerative diseases such as AD or PD (Williams-Gray et al., 2006; Dubois et al., 2007). Therefore, one can view episodic memory functioning as a highly sensitive indicator or seismograph of incipient brain pathology which manifests well before the full dimension of the disease becomes evident at the psychological and behavioral level.

### Mild cognitive impairment and Alzheimer’s disease

Presently, more than 24 million individuals across the world suffer from dementia and the majority of these cases is likely to be caused by AD. This high prevalence is predicted to double every 20 years because of the unprecedented level of aging in developed countries and the increased life-expectancy in the fast developing threshold countries (Ballard et al., 2011). The major histopathological hallmarks of AD include the loss of synapses, neurodegeneration, extracellular amyloid plaque deposits, and intracellular neurofibrillary tangles (Yankner, 1996a,b; Ballard et al., 2011), affecting brain areas within the medial temporal lobe, such as the entorhinal cortex and the hippocampus (Schwindt and Black, 2009). AD is a progressive, irreversible, and severely disabling disorder of memory and cognition that inevitably results in the need of intensive care and death. Accordingly, the health-care costs associated with the disease are exceptional high. There is a preclinical period covering several years in both AD and other dementias, called MCI, during which early and mild cognitive deficits can be identified (Brand et al., 2003; Galluzzi and Frisoni, 2008; Bordet et al., 2010). On average 50% of the individuals with MCI will develop AD within a few years (Trojanowski et al., 2010). Cognitive decline in early stages of AD typically begins with deficits in episodic memory (Bäckman et al., 2004; Ringman, 2005; Sarazin and Dubois, 2005; Thomas-Anterion and Laurent, 2006; Bäckman and Small, 2007; Dubois et al., 2007; Small and Bäckman, 2007; Schwindt and Black, 2009).

Until to date, there is no definite diagnostic test available which could identify AD in living patients (Reitz et al., 2010). Furthermore, the establishment of diagnostic tools for AD is time-consuming and expensive. Imaging tests include the measurement of amyloid-beta burden with PET (Nordberg et al., 2010) or measurements of hippocampal and frontal cortex volume with MRI (Wagner, 2000). Other tests include the analysis of cerebrospinal fluid biomarkers, e.g., the concentration of Aβ-oligomerization, Aβ-fragments, or phosphorylated-Tau (Blennow, 2010a,b; van Rossum et al., 2010). Although, these measurements have a high predictive value for identifying individuals in the prodromal stage of AD among patients with MCI (Hampel et al., 2010), these tests are too time-consuming, invasive, and expensive for being adopted as a routine screening. Therefore, AD is often unrecognized or misdiagnosed in its early stages.

Currently, the patient populations used to test the efficiency of drugs to ameliorate AD symptoms or its pathology in clinical trials are either very heterogeneous, as in the case of individuals with MCI, or patients are in a progressed stadium of AD where the effect of the drug is limited by extensive neurodegeneration. In fact, the potential of pharmacotherapy to ameliorate symptoms and/or pathology in AD has not yet been fully explored because of the difficulty to identify patients in a prodromal stage of AD (Blennow, 2010a,b).

The cognitive tests currently used for the diagnosis of AD are unspecific and cannot exclude cognitive impairments induced by other diseases. Furthermore, these tests are not sensitive to very mild forms of cognitive impairment (Pike and Savage, 2008). Thus, the main problem is that by the time the patient is diagnosed with AD, the disease had been progressed to a stadium in which pharmacological, immuno- (Weiner and Frenkel, 2006), and gene-therapy treatments (Urbaniak-Hunter et al., 2010) have only limited effects because of the advanced neurodegeneration in the target structures such as the frontal cortex or the hippocampus.

Therefore, there is a need for the development of a new cognitive marker of the presymptomatic stage of AD based on a test of episodic memory functioning. With such a diagnostic episodic memory test at hand it might be possible to identify individuals with a presymptomatic stage of AD among individuals with MCI. It might then be possible to delay the onset of the disease or to decelerate its progression with the currently available anti-Alzheimer disease treatments (Di Stefano et al., 2011; Pettersson et al., 2011).

### Dissociative amnesia

Dissociative amnesia also known as functional or psychogenic amnesia is a disease condition characterized by a transient and selective inability to retrieve episodic and autobiographical memories after experiencing traumatic events that have been associated with intolerable high levels of enduring stress (Brandt and Van Gorp, 2006; Reinhold and Markowitsch, 2009). Patients with dissociative amnesia have generally no impairment in the formation and retrieval of new memories as in the case of organic anterograde amnesia. The transient episodic memory retrieval deficit can be confined to episodic memories of a certain age and usually includes the memory for the traumatic event. It is assumed that the inability to retrieve traumatic events serves as a protective mechanism that inhibits the conscious recollection of self-endangering memories (Kapur, 1991).

Imaging studies have revealed functional changes in the activity of the right temporal-frontal region and in the inferior-lateral prefrontal cortex in brains of patients with dissociative amnesia during the resting state (Brand et al., 2009). Furthermore, it has been proposed that the inability to retrieve episodic memories in patients with dissociative amnesia might be related to functional disturbances in the activity of the precuneus, the lateral parietal, the right dorsolateral, and polar prefrontal cortex (Markowitsch et al., 1997). There is also recent evidence suggesting that the inability to retrieve episodic memories in dissociative amnesia is correlated with functional alterations of left posterior parietal cortex (Arzy et al., 2011).

Dissociative amnesia is an example for the disruption of episodic memory function induced by extreme levels of emotional activation or stress that is enduring and uncontrollable. The case of dissociative amnesia suggests that extreme levels of emotional activation or stress do not interfere with the formation of durable episodic memories but rather with their accessibility during attempts of retrieval. With regard to the findings with dissociative amnesia, the relationship between emotional activation and the formation of durable episodic memories could be précised as follows: Too low levels of emotional activation are not suited to consolidate episodic memories into long-term memory, while extreme levels of emotional activation or stress (transiently) impair the retrieval of durable episodic memories. This hypothesis will be outlined in detail below.

## Assessing Episodic Memory

### Conventional measures

The pertinent literature indicates that even the measurement of human episodic-like memory (without an explicit test of episodic or autonoetic recollection) based on the rather narrow (initial) definition of Tulving (1972) as a memory system that receives and stores personal experiences in terms of what, where, and when information was and is a daunting task, with only very few successful approaches available (Dere et al., 2004, 2010). For example, due to the lack of a valid test, the utility of subtests of the Mini Mental Status Examination Test (Carcaillon et al., 2009) or the Wechsler Memory Scale (Humphreys et al., 2010) have been evaluated as tools to measure episodic memory or episodic-like memory. The subtests of the Wechsler Memory Scale only measures verbal and visual memory and not episodic memory or episodic-like memory as defined above. The Mini Mental Status Examination Test is used for assessing the severity of dementia, comprising items on semantic memory and executive functions. Thus, both tests are not suited to detect selective episodic memory or episodic-like memory impairments that are associated with, e.g., MCI.

Currently used more specific tests of episodic memory or episodic-like memory function either measure the learning and reproduction of study list items such as the California Verbal Learning Test (Woodard et al., 1999; Fine et al., 2008; Lekeu et al., 2010) or are based on verbal or written reports of autobiographical events, such as in the Autobiographical Interview (Gilboa et al., 2005; Dreyfus et al., 2010; Irish et al., 2011).

### Verbal learning tests

In the recognition test studies, healthy participants or patients are requested to discriminate previously studied word or picture items from novel items (e.g., California Verbal Learning Test, Grober and Buschke, 1987). In some studies the maintenance of the learned material due to rehearsal in-between the encoding and retrieval sessions is prevented by an intervening and distracting task. The Rey Auditory Verbal Learning Task is based on the interference induced by the consecutive learning of two word lists and the immediate and delayed recall of the first list (Lezak, 1995).

The problem with verbal learning tests is that they do not explicitly measure whether the spatial and temporal context of the learning event is indeed recalled and often there is no spatial and temporal component implicated in the way the verbal memory is tested. Although, one can certainly assume that healthy adults are potentially able to remember where and when the recognized items have been presented, this is not sure in patient populations so that this ability has to be measured directly. Therefore, simple recognition tests cannot be used for episodic memory or episodic-like memory assessment in patient populations. Another problem associated with simple word list or picture recognition tests is that the participant or patient is instructed to memorize the learning material. This instruction is very likely to activate semantic instead of episodic memory systems for learning and consolidation of the learning material.

### The autobiographical interview

Questionnaires for autobiographical or important life events have also been used to asses episodic memory function (Levine et al., 2002; Piolino et al., 2009; Dreyfus et al., 2010). Kopelman et al. (1989), have developed a semi-structured interview schedule, that assesses the recall of autobiographical incidents and of “personal semantic” memory across three broad time periods termed: “childhood,” “early adult life,” and “recent” events/information. With the term “personal semantic” memory, they refer to factual knowledge about a person’s own past (e.g., addresses where lived, names of teachers/friend, colleagues at work, etc.). Thus, individuals are asked to recall a number of events from different periods of their life such as early childhood etc. or to recall a series of positive, negative, and neutral episodes from their lives. Usually, the participants or patients are asked to describe complete episodes that happened at a certain time and place. The verbal or written reports on events are then scored according to the frequency of episodic and non-episodic detail categories given. Episodic details refer to event, time, place, perceptual, thought, and emotion information (Kopelman et al., 1989). The general idea is that the fewer episodic details remembered for a given life event, the stronger the impairment in episodic memory functioning. However, there are a number of problems associated with this form of episodic memory assessment.

Generally, autobiographical memories are likely to be retrieved and narrated many times and due to their constructive and dynamic nature there might be changes in the contents and stability of a memory across time (e.g., due to reconsolidation and interference phenomena) (Schacter et al., 1998; Walker et al., 2003; Earles et al., 2008). Therefore, it is not always clear what type of memory (e.g., episodic or semantic memory) is actually measured by autobiographical memory tests. To account for this problem, tests have been designed that aim to distinguish between episodic and semantic components of autobiographical memory by scoring the spatio-temporal uniqueness of events (Levine et al., 2002; Piolino et al., 2009). Another problem is that episodic memory or episodic-like memory function is obviously judged by the assessment of the episodic memories that *can* be retrieved.

In early stages of neurodegenerative diseases such as MCI and AD autobiographical memories for personal life events which have been established decades before the onset of memory complaints are, although still accessible (Squire and Alvarez, 1995), possibly not completely normal. It has been shown that such autobiographical memories are overgeneralized, contain less specificity, and are less vivid (Murphy et al., 2008; Donix et al., 2010; Martinelli et al., 2013). Nevertheless, it is reasonable to assume that any impairment in episodic memory or episodic-like memory function should also impair the ability to encode and consolidate new or more recently formed episodic memories. These difficulties should manifest themselves as an inability to form novel episodic memories possibly independent of, or in addition to, changes in the ability retrieve episodic memories. Although the recollection of overgeneralized autobiographical memories for personal life events might be a hint for disturbed episodic memory function, reports on more recent events are probably more valid measures of the current status of episodic memory function.

Furthermore, the performance is also dependent on factors such as verbal competence, the ability and disposition to express feelings and emotions, the level of education, and pre-morbid verbal intelligence.

In conclusion, there is still a need for a valid standardized test to induce episodic-like memories and measure their retrieval. The arguments raised above suggest that it might be better to measure novel episodic memories rather than just ask the participant to elaborate on memories from the individual’s personal history, without having the option to control the exact circumstances and the time of episodic memory formation.

## Animal Research on Episodic-Like Memory

Animal research with rats and mice allows the study of the neurobiological mechanisms that might underlie episodic-like memory formation and retrieval with methods that are not available in human research. The animal research on episodic-like memory has its origins in now classic experiments on food-hoarding behavior in birds performed by Nicola Clayton and Anthony Dickinson (Salwiczek et al., 2009, for review). This line of research will be briefly discussed in the following paragraph. Thereafter we will discuss the importance of using one-trial memory paradigms (contextual fear conditioning and novelty-preference) which bear the unique capacity of being translatable to human episodic-like memory research.

### Food-hoarding paradigms

The first evidence for episodic-like memory in animals was provided by Clayton and Dickinson (1998) showing that birds (scrub jays) are able to remember where and when they have cached a particular food item. In the studies that followed, it was reported that corvids are also able to use tools (Cheke et al., 2011), show object permanence (Salwiczek et al., 2009), plan for the future (Raby et al., 2007), and might have a theory of mind (Emery and Clayton, 2004; Dally et al., 2006; Grodzinski and Clayton, 2010; but see Roberts, 2002; Dere et al., 2008; Rattenborg and Martinez-Gonzalez, 2011 for critical discussion).

In fact, all these remarkable abilities of corvids have been demonstrated with behavioral paradigms that are in one or the other way related to food caching and retrieval behavior which is very likely to be a genetically fixed instinctive behavior. In other words, all of these abilities might be restricted to a narrow class of content or what information. In contrast, the human episodic memory is not restricted to a specific type of events or stimuli. Therefore, it is indispensable to show that these birds are also able to perform all of these sophisticated tasks with stimuli other than food, such as the objects which do not have a biologically significant meaning to the animals (despite being of course novel stimuli) used in the episodic-like memory task developed by Dere et al. (2005a).

### Contextual fear conditioning

In the one-trial contextual fear paradigm, the animal receives a food shock upon the transition from a brightly lit large chamber into a smaller dark one (Sara et al., 1975; Ebenezer, 1988; Rudy and Sutherland, 1995; Rudy et al., 2004). If the animal is reintroduced to the big chamber, e.g., after a delay of 24 h, one finds that the animal avoids the normally preferred dark chamber. Thus, one-trial contextual fear conditioning can be described as a type of associative learning that is associated with a strong emotional arousal due to the sudden application of a painful stimulus in a specific spatial context. The formation of this memory seems to depend on hippocampal NMDA-receptors in the CA3 region (Cravens et al., 2006). It can be acquired in one trial, leads to a memory that is quite long-lasting (up to months), and is sensitive to hippocampal lesions (Anagnostaras et al., 2001; Lehmann et al., 2007). Therefore, there are some similarities between one-trial contextual fear conditioning and human episodic-like memory.

However, concerning the formation of episodic memory, the main problem that is associated with the contextual fear paradigm is that it does not measure whether the animals remember the temporal context of the aversive event (when information). Furthermore, human episodic memories are not restricted to fearful events, but also cover pleasant events (Ehrlichman and Halpern, 1988; Hamann et al., 1999). Nevertheless, the contextual fear conditioning paradigm has been useful to study the neurobiological mechanisms that might underlie one-trial spatial memory formation in rodents (Tronche et al., 2010; Li et al., 2011).

### The novelty-preference paradigm

The novelty-preference paradigm is based on the attraction of rats and mice by novel objects (Dere et al., 2007; Ennaceur, 2010; for a review see Dere et al., 2007). If rodents have the choice between an old and novel object they spent more time exploring the more interesting novel object suggesting that they remember the physical characteristics of the old object. The exploration of a novel object reflects approach behavior and likely to be motivated by an emotional activation that is rather pleasant to the animal and triggers episodic-like memory formation (reviewed in Dere et al., 2010). However, rodents prefer old objects when they have been placed in novel locations which render these objects more interesting as compared to old objects that are placed in a familiar location, which is taken as evidence for spatial object memory (Assini et al., 2009). In addition, rodents prefer objects which they have not seen very recently over those they have investigated more recently, which is taken as a measure for temporal order memory (Mitchell and Laiacona, 1998). By combining these different versions of the novelty-preference paradigm Dere et al. (2005a,b) tested whether rodents are able to remember specific events in terms of what happened where and when.

### The episodic-like memory test

The episodic-like memory test for rats and mice (Dere et al., 2005a; Kart-Teke et al., 2006) allows the simultaneous measurement of the memory for the temporal order of objects presented within an open-field as well as the spatial positions in which these objects have been encountered. The test is also able to detect whether what, where, and when information has been integrated into a unified multi-dimensional episodic memory (Kart-Teke et al., 2006; Zlomuzica et al., 2007). In brief, each animal receives two sample trials with four identical copies of a novel object and an inter-trial interval of 50 min followed by a test trial in which two objects from the first sample trial (old objects) are presented together with two objects known from the second sample trial (recent objects). During the test trial one of the old and one of the recent objects are displaced to a novel location. It has been demonstrated that rats and mice are able to associate object-, spatial-, and temporal information after a single exposure to such stimulus constellations (Dere et al., 2005a,b, 2007). This remarkable ability is in accord with the concept of episodic-like memory and can be further classified as a kind of one-trial contextual learning (Kart-Teke et al., 2006; Zlomuzica et al., 2008).

## Behavioral Criteria for the Measurement of Episodic-Like Memory

Findings from animal research were useful for the better understanding and definition of the conditions that might lead to episodic-like memory formation and its retrieval as well as for the generation of objective behavioral criteria by which the different features of episodic-like memory can be operationalized experimentally and can be assessed in both animals and humans (Clayton and Dickinson, 1998; Dere et al., 2005a,b, 2006). Accordingly, it has been proposed that episodic-like memory and episodic memory formation is an automatic, one-trial learning process based on the principles of one-trial contextual conditioning that is induced by a strong pleasant or aversive emotional activation (Dere et al., 2010). The generation of an episodic-like memory itself can be inferred from behavioral expressions referring to the content (what happened), place (where did it happen), and temporal context (in terms of the sequence of events attended) of personally experienced emotional events (Dere et al., 2006, 2008, 2010). Based on this behavioral definition of episodic-like memory a novel paradigm to induce and measure this type of memory non-verbally via quantifiable and reproducible motor responses in humans has been devised that will be presented below (Pause et al., 2010).

### Emotions trigger episodic memory formation

In order to measure novel episodic or episodic-like memories in the laboratory for research and diagnostic purposes it is necessary to define the properties of the events which induce episodic memory formation. Usually, only a subset of events that have occurred in the course of a day can be recalled after a few hours and the percentage of events that can be recalled decreases further if the delay between encoding and recall is extended to days, weeks, months, and years. The important question here is why are some events only stored for a few hours in the episodic memory system while others are stored permanently.

According to a distinction in memory duration, the classic test of episodic memory “please describe your breakfast this morning” would be a test of short-durable episodic memory, because it is obvious that the question “please describe your breakfast on Monday the week before last” will not yield a vivid description of this event. In contrast, the event of a car accident that occurred later on that particular Monday morning when the person was for example driving to its work place will be permanently remembered in great detail. It has been proposed that the impact of the trigger for episodic memory formation that decides for how long an event is stored in episodic memory is dependent on the emotional arousal that is associated with that event (Libkuman et al., 2004; Dere et al., 2010). Here, the rule might be that the stronger the emotional activation, the longer the durability of the episodic memory. Of course, this does not affect the fact that the durability and content of memories can also be modulated by factors such as rehearsal or the number of previous recalls of the memory (Zimmer et al., 2003; McKenzie and Eichenbaum, 2011). Furthermore, it is important to note that extreme emotional activation (e.g., stress) can disrupt episodic memory function similar to other types of memory. An example for such stress-induced impairment of episodic memory function is discussed under the section dealing with dissociative amnesia.

Furthermore, it is possible that the relationship between episodic memory performance and the degree of emotional activation during the encoding of episodic information has the form of an inverted U-shaped curve as it has been described for the dose-response function between orally administered cortisol and explicit memory performance in humans (Abercrombie et al., 2003; Baldi and Bucherelli, 2005). This assumption implicates that excessive emotional activation similar to insufficient emotional activation would impair episodic memory formation. One example for impaired episodic memory formation after excessive emotional activation is that of a trauma memory which can be either fragmentarily or incorrectly remembered (Bower and Sivers, 1998; van der Hart and Nijenhuis, 2001; Brewin, 2011). Moreover, emotional arousal might not be only a trigger for episodic memory formation but might also play a role in binding of different features of an event into a unified episodic memory (Nashiro and Mather, 2011).

One prediction of the necessity of emotional arousal for the establishment of long-durable episodic memories would be that bilateral amygdala damage impairs episodic memory function. In this regard, it has been proposed that the amygdala contributes to the integration of emotion, perception, and the memory for past autobiographical events, plays an integral part in the establishment and maintenance of an integrated self, and provides retrieval cues during the memory search for emotionally significant events (Markowitsch and Staniloiu, 2011). Evidence for an implication of the amygdala for the encoding and retrieval of episodic memories has been provided by the examination of patients with Urbach–Wiethe syndrome who suffer from relatively selective bilateral lesions of the amygdala (Hurlemann et al., 2007). In fact, it has been reported that Urbach–Wiethe patients show an impairment of the memory for autobiographic episodes but not for autobiographic facts (Wiest et al., 2006). Furthermore, it has been reported that a 54-year-old woman with bilateral damage to the amygdala showed impaired episodic memory performance for stimuli that elicited an arousal response (taboo words) but not for emotional stimuli which did not elicit an arousal response (Phelps et al., 1998).

### The temporal component of episodic memory

A main difference between episodic and semantic memory is that the episodic memories have also a temporal connotation are time-dated or endowed with time-tags such as that something has happened “this morning” or “last summer” (Tulving, 2001, but see also Klein, 2013 this issue for a different view). However, in humans the perception of time and the assessment of the duration of events are not represented on a linear time scale (Pöppel, 1997; Roberts, 2002). In this regard, Friedman (1996) speaks of a “chronological illusion,” and proposes that the memory for time is rather reconstructive and inferential in nature.

We have proposed that temporal information is either stored as succession or order information relative to other events already stored in episodic memory or is reconstructed during the recall processes using anchor events (events for which an episodic memory exists) (Dere et al., 2006). It is reasonable to assume that the temporal context of a particular event is either encoded in relation to, or inferred from its occurrence before or after, other “anchor events.” One of which is proximal in terms of just preceding or following the event to be temporally specified, whereas the other is more distal that is the anchor event stands at the beginning or end of a larger sequence of events centered by the event to be temporally specified. The when component of an episodic memory can thus be operationalized by successively presenting two or more distinct events and by probing whether the participants are able to remember their order of occurrence (Dere et al., 2006, 2008).

### The retrieval of episodic memory

One difficulty that is associated with the traditional concept of episodic memory is that they do not specify the circumstances that lead to the retrieval of episodic memories besides postulating that it should be a “free recall” instead of a “cued” recall (Yonelinas, 2002). This assumption might not be correct since it is unlikely that any memory retrieval either episodic or semantic occurs without a cue stimulus which triggers memory retrieval (Dere et al., 2004; McCabe et al., 2011; Unsworth et al., 2011). There must be a cue stimulus present either produced internally (e.g., a thought) or externally, within the environment that triggers the memory retrieval (Light and Albertson, 1989). Otherwise our episodic and semantic memories would be retrieved in an uncontrolled and highly disturbing manner.

Recently, it could be demonstrated that odors are highly valid stimuli cuing episodic or autobiographic memory contents (Aggleton and Waskett, 1999; Saive et al., 2013). Odor evoked memories are experienced as more emotional and are associated with feelings of being brought back in time to the occurrence of the event (Masaoka et al., 2012; Arshamian et al., 2013). The potency of odors to evoke highly vivid episodic memories has been discussed to be due to the emotional nature of olfactory stimuli (Pause et al., 2008; see Laudien et al., 2008; Adolph and Pause, 2012).

The new concept of episodic memory presented here holds that episodic memory formation is based on principles similar to one-trial contextual conditioning and that its retrieval depends on the presence of a cue or conditioned stimulus. This concept is at variance with the traditional distinction between declarative and non-declarative memory (Squire, 2004) in which episodic memory is generally attributed to declarative memory, while classical conditioning, including contextual conditioning, is attributed to non-declarative memory. It seems that it is time to revise this useful nomenclature of long-term memory systems to better reflect the novel insights gained into the nature of episodic memory.

## Promising Novel Paradigms

### Spatial and temporal order memory for emotional pictures

In a reverse translational approach by Pause et al. the rationale and principles of the episodic-like memory test for rodents (Dere et al., 2005a,b; Kart-Teke et al., 2006) have been adapted to humans. Pause et al. (2010) developed a paradigm which measures the spatio-temporal memory for emotional and neutral pictures presented on an eight-quadrant computer-screen task. Visual stimuli were hidden in four out of eight quadrants on a computer-screen. Each of these quadrants could be highlighted by pushing a corresponding button on a keyboard which either revealed a visual stimulus for 100 ms or remained black. Participants had to remember on which occasion (when) and at which position (where) a specific picture (what) has been encountered. It was investigated whether exploratory button presses can be used as a non-verbal behavioral expression of what, where, and when memory. Verbal what, where, and when memory performance was measured by a standardized interview.

Pause et al. (2010) also examined whether the variation of the visual stimuli in terms of concreteness and emotional content would have an effect on what, where, and when memory performance or quadrants exploration. In the verbal test, healthy adult participants were able to recollect the details and the spatio-temporal context of visual stimuli with combinations of high or low concreteness and neutral or emotional content after retention delays up to 72 h. The number of button presses to each of the four quadrants containing visual stimuli was positively correlated with verbal what, where, and when memory performance.

These results demonstrate that what, where, and when memory can be induced experimentally and can be measured by motor behavior which can serve as a non-verbal correlate of episodic memory performance (Pause et al., 2010). Compared to classical methods this novel paradigm is certainly an improvement as it measures novel long-term memories for what, where, and when information. However, since the participants were instructed to memorize the learning material, the task might not only have involved episodic memory mechanisms. Therefore, a slightly different version of this paradigm has been applied in young and elderly participants (Kinugawa et al., 2013, this issue). Here, the participants were not informed to memorize the test items and the memory retrieval was not foreseeable for the participants. Furthermore, explicit memory performance was tested already 2.5 h after the initial learning phase. It was found that the young group (age: 21–45) had significantly higher episodic memory scores as compared to both the middle (age: 48–62) and aged group (age: 71–83). The latter two groups performed not significantly different from each other. The results suggest that this novel test of what, where, and when memory that measures the core components of an memory for event, spatial, and temporal information and in addition is likely to probe the ability to form new episodic memories is suited to detect age-related impairments in what, where, and when memory performance. Due to its simplicity, its one-trial nature and the option of a non-verbal measurement this task could be probably adapted to clinical populations.

### The Kensinger episodic-like memory task

In a recent fMRI study by Kensinger et al. (2011) on the role of the amygdala for the encoding of episodic-like memories the participants studied four sets of emotional pictures. It was found that amygdala activity was correlated with item-specific details of the learning material used and the subjective assessment of memory vividness, but not with the diversity of episodic details remembered (Kensinger et al., 2011). In this study, each picture was presented in one out of four quadrants of a monitor screen. On the retention test performed 30 min after the presentation of a total of four picture sets, verbal labels corresponding to the pictures viewed were presented. This approach aims to induce and measure a memory for what, where, and when. The what and where components have been operationalized by different pictures presented in different quadrants on a monitor screen. The temporal component of episodic-like memory was operationalized by the four different sets of pictures. In general the task was associated with a rather high memory load. The participants had to memorize four sets with a total of 30 pictures and as well as their positions on the screen. Furthermore, the task performance certainly depended on pre-morbid learning abilities and the susceptibility to interference. For these reasons the task might be too complicated to be performed with individuals suffering, e.g., from MCI. Furthermore, a retention delay of only 30 min might not fall into the range of long-term episodic memory and retention performance might involve short-term memory phenomena such as recency effects. The combination of emotional pictures with spatial and temporal information is certainly a useful approach to identify the neuroanatomical substrates of episodic-like memory in humans.

### Virtual reality-based tasks

Another reverse translational approach aims to induce and measure core elements of episodic-like memory such as spatial and temporal order memory in humans with a navigational “starmaze” task, initially developed to measure egocentric and allocentric spatial orientation in rodents (Rondi-Reig et al., 2006; Fouquet et al., 2011). Here the individual’s ability to remember a sequential order of choices during spatial navigation in a virtual reality environment is measured (Fouquet et al., 2010). The participant has to learn to reach a goal using the most direct path. During the multiple acquisition trials, the individuals can use allocentric (spatial reference memory) or egocentric (temporal order based) strategies to solve the task. Fouquet et al. (2010) propose that the use of the egocentric strategy to perform this task involves an “active, flexible, and complex integration of past and current states” which is a feature shared by episodic memory. Following this rationale it can be predicted that an individual with impaired episodic memory function would be forced to choose the allocentric strategy to learn the path through the virtual environment.

Allocentric orientation in a novel environment is a function which is critically dependent on the integrity of the hippocampal formation in rodents and humans. Furthermore, the hippocampus is also indispensable for the integration of spatial and temporal information into a unified episodic memory (Ranganath, 2010). In fact, animal studies have shown that rodents with hippocampal lesions shift to egocentric striatum-based strategies to solve spatial tasks and that healthy controls spontaneously use allocentric strategies and only shift to egocentric strategies if the former is not successful (Oliveira et al., 1997; DeCoteau and Kesner, 2000; Dere et al., 2001). Nevertheless, this approach is interesting and certainly worth of being evaluated with patient populations.

Yet another approach to measure episodic memory function using virtual reality technology has been recently communicated by Plancher et al. (2012a). In this study the authors argue that virtual reality-based tests allow to investigate episodic memory profiles in an ecological fashion, including the assessment of the memory for central and perceptual details, spatio-temporal contextual elements, and binding of these multi-dimensional information. The authors tested older individuals, patients with MCI as well as patients with early stages of AD. These groups were tested under active and passive exploration conditions and were instructed to memorize as many information as possible as well as all spatio-temporal information that was associated with detail information. As expected the performance of patients with AD was inferior to that of the patients with MCI and healthy older individuals. The authors found that spatial allocentric memory assessments discriminated between patients with MCI from controls and that active exploration was associated with better performance in all of the three groups.

Additional studies by this and other groups have further substantiated the important influence of enactment, exercise, and active engagement of subjects in the encoding phase (Madan and Singhal, 2012; Plancher et al., 2012b). The theoretical background of this line of research is derived from the concept of embodied cognition that holds that physical properties of the human body, particularly the perceptual and motor systems, modulate learning and memory formation (Madan and Singhal, 2012).

In conclusion virtual reality and enactment-based tests of episodic-like memory might allow measuring this function in a more ecological fashion. However, it is also important to avoid explicit instructions asking the participants to memorize the information presented, including precise instructions on what kind of information should be memorized. This kind of instruction might prompt the participants to use semantic learning strategies rather than incidental episodic memory encoding. It is important to note that although the recollection of episodic memories is associated with autonoetic awareness, its encoding is rather automatic and effortless (Zentall et al., 2008; Dere et al., 2010). Generally, any assessment of episodic-like and episodic memory should be unexpected.

## Basic Criteria for Episodic-Like Memory Test Development

Although, the above reviewed novel approaches to measure episodic-like memory have moved the field forward toward a valid and reliable test of episodic memory (besides of not probing autonoetic recollection) all of them have their pitfalls and are not easily applicable to patient populations. Similarly, animal research has led to the definition of behavioral criteria that are considered important for the measurement of episodic-like memory in animals and also for the measurement of episodic-like memory in humans (Griffiths and Clayton, 2001; Dere et al., 2008).

In the following we will outline seven basic criteria which should be met by a test of episodic-like memory. These seven criteria might facilitate the development of routine diagnostic tests of episodic-like memory for patient populations and tests to be utilized for the investigation of the neurobiological and electrophysiological substrates of episodic memory in healthy individuals.

*First criterion*, in order to be able to control the correctness of the episodic-like memory retrieved and also to control the time of encoding of these events, as well as to have the option to manipulate experimental variables that would affect episodic memory formation in healthy and patient populations differently, it is necessary to induce the episodic memories in the laboratory.

*Second criterion*, the participants of an experimental investigation or patients to be tested for episodic-like memory integrity should not receive any explicit instruction to memorize the episodic information. Otherwise, the episodic information might be acquired and retained by semantic memory systems, e.g., by rehearsing the episodic information until it is consolidated using a semantic memory system.

*Third criterion*, since long-durable episodic-like memory which are permanently stored are generally related to important life events which have been accompanied and probably induced by a relatively strong (but not excess, see above) emotional activation, the episodic information to be retained needs to have an emotional valence and should be associated with emotional arousal to be demonstrated at the level of physiological stress responses or autonomic activation associated with pleasant events.

*Fourth criterion*, the induction of the episodic-like memory must be per definition a one-trial learning event. The acquisition of episodic information presented repeatedly is always mediated through a semantic memory system.

*Fifth criterion*, the episodic information to be induced, retained and retrieved must include what, where, and when information. Episodic-like memory assessment has to include the measurement of the ability to remember what, where, and when information.

*Sixth criterion*, the memory test has to be unexpected and only the first recall is a valid measure of episodic-like memory performance. Generally, the possibility to perform re-tests or multiple assessments of the same individual with the same episodic memory test is therefore very limited.

*Seventh criterion*, the storage of long-durable episodic-like memories requires memory consolidation which requires the activation of specific genes and *de novo* synthesis of proteins (Abel and Lattal, 2001; Igaz et al., 2002). Drugs that block protein synthesis such as antibiotics have amnesic effects when administered immediately after the acquisition of new information (Schafe et al., 1999; Díaz-Trujillo et al., 2009) but not after delays of 60 min or more (Fulton et al., 2005; Rossato et al., 2007; Lima et al., 2009). Therefore, the memory test has to be performed after a retention interval of at least 60 min after presentation of the episodic information to be considered a fully consolidated long-term memory. Tests which adhere to these requirements can be considered as measuring episodic-like memory in a valid and reliable way.

## Basic Criteria for a Test of Episodic Memory

A valid test of human episodic memory should include the seven criteria defined above to qualify as a test of episodic-like memory and should also measure phenomenological aspects of episodic memory such as autonoetic awareness, self, subjective sense of time. This could be potentially done with a separate tests performed after the episodic-like memory test. In the past it has been attempted to measure autonoetic awareness with the remember/know procedure derived from the dual-process signal-detection theory (Yonelinas, 2002). With this method individuals are asked to learn a list of items and thereafter presented with items form the list and novel items. If the subject recognizes an item the participant is asked to state whether it “remembers” that the item was on the list or whether he/or she just “knows” that the item was on the list because of a feeling of familiarity. It is obvious that this procedure is not suited to capture the richness and complexity of Tulving’s understanding of episodic memory (Tulving, 2002) “*Episodic memory is a recently evolved, late-developing, and early-deteriorating past-oriented memory system, more vulnerable than other memory systems to neuronal dysfunction, and probably unique to humans. It makes possible mental time travel through subjective time, from the present to the past, thus allowing one to re-experience, through autonoetic awareness, one’s own previous experiences. Its operations require, but go beyond, the semantic memory system. Retrieving information from episodic memory (remembering or conscious recollection) is contingent on the establishment of a special mental set, dubbed episodic “retrieval mode.” Episodic memory is subserved by a widely distributed network of cortical and subcortical brain regions that overlaps with but also extends beyond the networks subserving other memory systems. The essence of episodic memory lies in the conjunction of three concepts – self, autonoetic awareness, and subjectively sensed time*.”

## Conclusion

Although, one can note some progress toward the goal of a valid and reliable test of episodic-like memory that can be utilized in clinical settings for diagnostic purposes the currently available approaches still have to be optimized and modified before they can be possibly adapted for patient populations. A valid test of episodic memory that also includes the phenomenological aspects of episodic memory is still lacking. The above criteria should help to approach the optimal solution more directly. It is hoped that this review will sensitize clinicians and researchers in the field of episodic memory to critically re-evaluate their current methods and tools to measure episodic-like or episodic memory and will induce a lively discussion concerning the urgent need to develop novel and better paradigms to measure episodic memory.

## Conflict of Interest Statement

The authors declare that the research was conducted in the absence of any commercial or financial relationships that could be construed as a potential conflict of interest.
